# Genome-Wide Association Study of Motor Coordination

**DOI:** 10.3389/fnhum.2021.669902

**Published:** 2021-06-09

**Authors:** Hayley S. Mountford, Amanda Hill, Anna L. Barnett, Dianne F. Newbury

**Affiliations:** ^1^Department of Biological and Medical Sciences, Faculty of Health and Life Sciences, Oxford Brookes University, Oxford, United Kingdom; ^2^Population Health Sciences, Bristol Medical School, University of Bristol, Bristol, United Kingdom; ^3^Centre for Psychological Research, Faculty of Health and Life Sciences, Oxford Brookes University, Oxford, United Kingdom

**Keywords:** coordination, development, dyspraxia, neurodevelopment, GWAS, ALSPAC, developmental coordination disorder, motor coordination

## Abstract

The ability to finely control our movement is key to achieving many of the educational milestones and life-skills we develop throughout our lives. Despite the centrality of coordination to early development, there is a vast gap in our understanding of the underlying biology. Like most complex traits, both genetics and environment influence motor coordination, however, the specific genes, early environmental risk factors and molecular pathways are unknown. Previous studies have shown that about 5% of school-age children experience unexplained difficulties with motor coordination. These children are said to have Developmental Coordination Disorder (DCD). For children with DCD, these motor coordination difficulties significantly impact their everyday life and learning. DCD is associated with poorer academic achievement, reduced quality of life, it can constrain career opportunities and increase the risk of mental health issues in adulthood. Despite the high prevalence of coordination difficulties, many children remain undiagnosed by healthcare professionals. Compounding under-diagnosis in the clinic, research into the etiology of DCD is severely underrepresented in the literature. Here we present the first genome-wide association study to examine the genetic basis of early motor coordination in the context of motor difficulties. Using data from the Avon Longitudinal Study of Parents and Children we generate a derived measure of motor coordination from four components of the Movement Assessment Battery for Children, providing an overall measure of coordination across the full range of ability. We perform the first genome-wide association analysis focused on motor coordination (*N* = 4542). No single nucleotide polymorphisms (SNPs) met the threshold for genome-wide significance, however, 59 SNPs showed suggestive associations. Three regions contained multiple suggestively associated SNPs, within five preliminary candidate genes: *IQSEC1*, *LRCC1*, *SYNJ2B2*, *ADAM20*, and *ADAM21*. Association to the gene *IQSEC1* suggests a potential link to axon guidance and dendritic projection processes as a potential underlying mechanism of motor coordination difficulties. This represents an interesting potential mechanism, and whilst further validation is essential, it generates a direct window into the biology of motor coordination difficulties. This research has identified potential biological drivers of DCD, a first step towards understanding this common, yet neglected neurodevelopmental disorder.

## Introduction

Developmental coordination disorder (DCD) is a neurodevelopmental condition defined by the DSM-5 as a severe impairment of motor skills, usually presenting in early childhood, and in the absence of any other explanatory factor such as a known neurological disorder (e.g., cerebral palsy, acquired brain injury, visual impairment, or intellectual disability) (American Psychiatric Association, 2013).

Developmental coordination disorder is thought to affect approximately 5% of 7–8-year-old children (American Psychiatric Association, 2013), although the estimates vary considerably between study populations and methodological approaches (Wright and Sugden, 1996; Tsiotra et al., 2006; Lingam et al., 2009). In practice, this means there are one or two children with DCD in every school class. Despite this high prevalence and official recognition as a neurodevelopmental disorder in the DSM-IV and -5, DCD remains chronically underdiagnosed by healthcare professionals (Blank et al., 2019). DCD is similarly underrepresented in the research literature, with ten-fold fewer research articles published on DCD than dyslexia between 1985 and 2009 (Bishop, 2010).

Children with DCD have difficulty acquiring fine and/or gross motor skills, making learning age-appropriate activities such as riding a bicycle or catching a ball extremely challenging, even given opportunity to practice. They are more likely to struggle with daily tasks, academically and socially, and are more likely to have overall poorer quality of life in adulthood (Stephenson and Chesson, 2008; Summers et al., 2008; Harrowell et al., 2018; Cleaton et al., 2019). These motor difficulties initially manifest in early childhood and have been shown to persist into adolescence in approximately half of individuals (Losse et al., 1991; Cantell et al., 1994) and a similar proportion continue to be affected into adulthood (Kirby et al., 2008, 2011). There is a current absence of longitudinal data to understand whether the proportion of individuals who improve is a result of access to intervention, developing their own coping strategies, through extensive and deliberate practice or through some other mechanism (Kirby et al., 2011). Therefore, the impact of DCD persisting into adult life is likely to be underestimated.

Many children receive their initial referral for motor function assessment because of their poor handwriting (Barnett and Prunty, 2020). This is when coordination problems are often first noticed in school and begin to affect their academic achievement. Poor academic outcomes are reported in children with DCD. A recent study found that individuals with DCD were less likely to finish school with 5 or more GCSEs (OR 0.27, 95% CI 0.21–0.34), placing them at a considerable disadvantage compared to other students (Harrowell et al., 2018).

Children with DCD require substantial extra support to successfully perform daily activities, as they often find basic tasks such as eating, washing, or cleaning their teeth extremely challenging (Summers et al., 2008). This extra support impacts upon family quality of life and can have a substantial financial and time cost to their carers (Cleaton et al., 2019). Further contributing to this, behavioral problems and difficulties with social interactions can make these relationships more challenging, with these problems often persisting into adulthood (Stephenson and Chesson, 2008).

Co-occurrence with other neurodevelopmental conditions is extremely common, particularly with attention deficit hyperactivity disorder (ADHD) and developmental language disorder (DLD) (Lingam et al., 2010). The reason for these co-occurring conditions is unknown; whether they are due to shared genetic and/or environmental risk factors, or perhaps that one may act as a risk factor for another. Anxiety and depression are commonly associated with DCD, placing individuals at a greater risk of a lifetime of increased vulnerability to mental health issues (Kirby et al., 2008). Individuals with DCD are less likely to participate in physical activities (Cantell et al., 1994) and have a higher chance of becoming overweight (Cairney et al., 2005), increasing their long-term risk of developing obesity related health problems.

The underlying etiology of DCD is multifactorial, and both environmental and genetic factors are thought to play a role. One major risk factor that has been repeatedly associated with DCD risk is premature birth (and therefore low birth weight) (Lingam et al., 2009; Larsen et al., 2013). Similarly, low socio-economic status (SES) has been shown to correlate with increased risk of DCD (Lingam et al., 2009). While these environmental factors have been robustly associated with increased risk of DCD, the mechanism by which they act upon early motor development remains unknown.

It is also widely accepted that genetics play a major role in an individual’s risk for developing DCD, although no specific genes or molecular pathways have been reported, to date. Several twin studies have examined the heritability of DCD and revealed genetic contribution estimates from 0.44 (Moruzzi et al., 2010), through to 0.7 (Lichtenstein et al., 2010) and 0.8 (Martin et al., 2006). Although these studies are relatively small scale and vary in their inclusion criteria, they indicate a relatively high potential genetic contribution, particularly for a neurodevelopmental disorder.

Genetic investigations into the underlying cause of other neurodevelopmental disorders such as dyslexia and DLD have identified both Mendelian variants and complex genetic models of susceptibility to contribute to their genetic basis (Becker et al., 2017; Mountford and Newbury, 2017). Mendelian variants are usually single genetic variants that have a negative impact on their resulting protein, preventing it from functioning as it should. This type of Mendelian genetic variation generally results in specific (and often more severe) forms of neurodevelopmental disorders and are extremely rare.

Complex genetic models consider genetic susceptibility or risk conferred by genetic variants that are more commonly found in the general population and interact with environmental factors to increase overall risk of developing a condition. Environmental influences can also act as protective factors, such as participation in sporting activities. So far, other neurodevelopmental disorders have examples from both of these genetic models. It is therefore highly likely that DCD will have a similar genetic etiology combining rare Mendelian variants in some rare cases, but more commonly an overall complex genetic risk which is influenced by risk and protective environmental factors. It is likely to be genetically and environmentally multi-factorial, with many subtle influences.

There have only been a few studies into the genetic basis of DCD, and so far, no genes have been identified as causative. One study reported a large Canadian family where five of seven children and their mother had a diagnosis of DCD (Gaines et al., 2008). This inheritance pattern is strongly suggestive of a fully penetrant dominant Mendelian genetic variant, which has been inherited from the affected mother, however, no investigation into the underlying genetic cause was reported.

Copy number variations (CNVs), large insertions and deletions of regions of the genome, have also been implicated in DCD and other neurodevelopmental disorders such as autism spectrum disorder (ASD) (Sanders et al., 2015), intellectual disability (Coe et al., 2014) and ADHD (Lionel et al., 2011). These insertions or deletions of genetic material can be inherited or occur sporadically. CNVs occur as a normal part of our individual genomic variation, and we each carry about ten unique or very rare changes. The presence of some specific CNVs results in a clear syndrome, for example certain deletions carried on 16p11.2 (chromosome 16) cause a specific type of language impairment called childhood apraxia of speech (CAS) (Newbury et al., 2013; Fedorenko et al., 2016). More frequently, the effect of an individual CNV is difficult to determine, depending on which genes are contained within the deleted or duplicated region and how much it affects their ability to function. Perhaps surprisingly, the majority of CNVs have no clear role or effect on cell function and appear to be completely tolerated.

The presence of more and/or larger genomic regions, known as enrichment or increased burden of CNVs have also been detected in individuals with DLD when compared to controls with typical language development (Simpson et al., 2015; Kalnak et al., 2018). Individuals with a higher burden, i.e., more CNVs or larger regions of their genomes contained CNVs, tend towards a more severe phenotype. In all these CNV studies, the statistical differences are extremely subtle, and difficult to detect. This is further hindered by individual CNVs resulting in highly heterogeneous phenotypes, and even some of the most common and best understood show a varied phenotype (Mountford et al., 2020).

Enrichment of CNVs has been reported in individuals with DCD (Mosca et al., 2016). This provides a direct link between the overall number and size of copy number changes and DCD, as has been established in other neurodevelopmental disorders.

Also, in cases where individuals carried a pathogenic CNV known to cause other neurodevelopmental disorders (e.g., the 16p11.2 deletion which results in CAS), Cunningham et al. (2019) showed that carriers of these pathogenic CNVs were more likely to have coordination difficulties. While this does not demonstrate a clear association between specific CNVs and coordination, it suggests a link between known causes of neurodevelopmental disorders and motor function. Taken together, these two studies are suggestive of a role of CNVs in motor coordination, and an interesting avenue for further investigation.

For common genetic disorders, the first line of investigation usually comprises of a genome-wide association study (GWAS), in which common genetic variants are compared between unrelated cases and controls. These genetic variants are single base pair changes, called single nucleotide polymorphisms (SNPs), and represent common variation within the general population. Only one GWAS has so far been performed to look for regions of the genome which are commonly shared by individuals with DCD. The study looked at 890 individuals with a diagnosis of DCD co-occurring with ADHD, and did not find any variants that reached genome-wide significance, but did report an enrichment for common variants located within genes with a known neurological function (Fliers et al., 2012). A particularly interesting finding from this study, was that eight of the nine genes that contained variants of suggestive association, played a role in mechanisms of neurite outgrowth and muscle function. Although this is a small and underpowered study, they suggest that motor coordination difficulties with ADHD are associated with genes with both neurological and muscular functions, however, this is yet to be fully elucidated.

In a wider sense, the underlying systems through which DCD and early coordination difficulties manifest are unknown and remains a major unanswered question in the DCD field. The current literature tends towards the theory that subtle neurological changes in the brain, including the cerebellum, may underlie DCD, as opposed to motor or muscular function (Licari et al., 2015; Blank et al., 2019). A recent study used diffusion tensor imaging (DTI) to identify differences in white matter between children with DCD and neurotypical controls (Brown-Lum et al., 2020). They detected white matter differences in three pathways: the corticospinal tract, posterior thalamic radiation, and cerebellar pathways. All three white matter pathways are involved in motor or sensorimotor function, providing compelling evidence that differences in axonal development in these regions may underlie DCD. The identification of genes associated with motor coordination may help to further delineate the underlying etiology and has the potential to link neurological changes to gene function.

One approach to the specific investigation of a disorder in clinical cohorts is the investigation of underlying traits in the general population. This can help to increase sample size and identify underlying molecular mechanisms. Here, we use the Avon Longitudinal Study of Parents and Children (ALSPAC) population data set to perform the first quantitative GWAS of motor coordination in a population cohort. We use four measures from the Movement Assessment Battery for Children (MABC) (Henderson and Sugden, 1992), the gold-standard measure of motor coordination difficulties in the ALSPAC dataset. The ALSPAC cohort provides data on a subset of children across four of the individual MABC tasks: heel-to-toe walking, placing pegs, threading lace and throwing a bean bag into a box (Henderson and Sugden, 1992). Collectively, these test items represent the most robust measure of fine and gross coordination in children with associated genetic data. Lingam et al. (2009) used three of these measures in ALSPAC (heel-to-toe walking, placing pegs, throwing a bean bag) along with additional criteria (see “Discussion”) to estimate that 1.8% of children at 7 years old meet the diagnostic criteria for DCD, and an additional 2.23% have probable DCD. Although the full MABC has not been performed in this cohort, Lingam et al. (2009) showed that the three MABC tasks reported in their study provide a reliable measure of three main domains of motor coordination (balance, manual dexterity, and ball skills). Lingam et al. (2009) further showed that these tasks had concurrent validity with other coordination tests from their own measures and from other studies (Van Waelvelde et al., 2004). In the current study we strengthen the measure of motor coordination by supplementing the three MABC tasks with an additional manual dexterity task (“threading lace” from the MABC) also used by Lingam et al. (2009).

Here we report the first genes to be directly implicated in motor coordination in children, generating the first window into the genetic basis of DCD.

## Materials and Methods

### Ethical Approval

Ethical approval for this study was obtained from Oxford Brookes University Research Ethics Committee (UREC #191311).

Ethical approval for ALSPAC (B2341) was obtained from the ALSPAC Ethics and Law Committee and the Local Research Ethics Committees^1^. Informed consent for the use of data collected via questionnaires and clinics was obtained from participants following the recommendations of the ALSPAC Ethics and Law Committee at the time. Consent for biological samples has been collected in accordance with the Human Tissue Act (2004).

### Avon Longitudinal Study of Parents and Children Population Cohort

The study was performed using a large UK population cohort; the Avon Longitudinal Study of Parents and Children (ALSPAC) consisting of 14,541 pregnancies to mothers in the Avon region with anticipated delivery dates of between 1st April, 1991, and 31st December, 1992 (Boyd et al., 2013; Fraser et al., 2013). Of these initial pregnancies, there were a total of 14,676 fetuses, resulting in 14,062 live births and 13,988 children who were alive at 1 year of age.

Avon Longitudinal Study of Parents and Children offers a broad range of developmental phenotype measures spanning the participants’ lives, including measures of gross and fine motor skills. Please note that the study website contains details of all the data that are available through a fully searchable data dictionary and variable search tool http://www.bristol.ac.uk/alspac/researchers/our-data/

A subset of children (*N* = 8,365) was genotyped by ALSPAC using Illumina Human Hap 550-quad arrays which allows the direct characterization of more than half a million common European genetic variants across the Human genome. These data were jointly phased using SHAPEIT2 (Delaneau et al., 2013), which uses relationship information to improve phasing accuracy, and imputed to the 1,000 Genomes v1.3. This imputation phase allows the prediction of uncharacterized variation using genetic data from unrelated individuals. The imputation dataset was filtered to include SNPs with an imputation quality score >0.8 (i.e., those with high confidence genotype calls).

Children were excluded from the current study if there were missing data from one of the four measures of coordination from the MABC (Henderson and Sugden, 1992); heel-to-toe walking (F7CR015), placing pegs with preferred hand (F7CR105), threading lace (F7CR211), and throwing a beanbag into a box (F7CR331). In total, data were available for 6500 children across all four measures.

DSM-IV/5 criteria for DCD include a qualification that children with a visual or physical disability which limits movement, or moderate intellectual disability should be excluded from a diagnosis of DCD. Children with a parental report of eyesight problems requiring special arrangements at school (age 7.5–KR566) or visual impairment (age 8.5–SA036A) were excluded. Similarly, parents who reported the child has physical problems requiring special arrangements at school (age 7.5–KR567), the child has ever had a physical disability at (age 8.5–SA037A), or the school reported the child has sensory and/or physical needs (visual impairment, hearing impairment, multi-sensory impairment, physical disability) requiring formalized government special education needs support at (age 11–12–PLASCC65) (*N* = 159) were excluded. Individuals were excluded if they answered yes to any of the five visual or physical exclusions, if the answers were inconsistent between measures across the time points, or if they were missing data across all three time points.

Finally, children were excluded if they showed evidence of moderate to severe intellectual disability (Weschler Intelligence Score for Children III–Full IQ ≤ 50 at age 8.5–F8WS112) (*N* = 669). Children with missing IQ data were excluded unless they scored above expected (level 5) in the nationally administered key stage 3 (KS3) assessments at age 13–14 in English (ks3_leve), math (ks3_levm), and science (ks3_levs) and were not reported as ever needing provisions for special educational support (age 7.5–KR561, age 8.5–SA030, and age 11–12–PLASCC40). We could confidently conclude that these children did not have moderate intellectual disability. Individuals missing IQ, KS3, and special educational support provision data were excluded.

Individuals were further excluded from this dataset if they did not have imputed genetic data available (*N* = 1097). Finally, to ensure that all children in the cohort are unrelated, in the case of twins one twin from each pair was removed from analyses (*N* = 33). The final cohort consisted of 4,542 children (2183M, 2359F).

### Phenotype and Derived Measures

**FIGURE 1:**
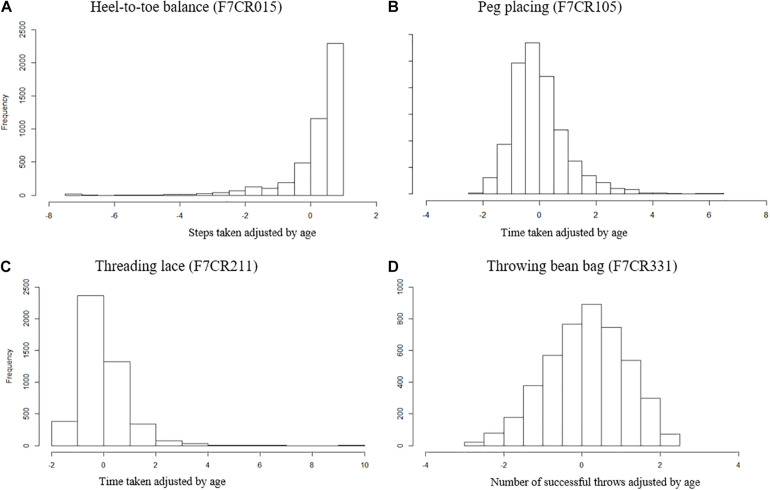
Distribution of MABC test items age adjusted. **(A)** Heel-to-toe walking (F7CR015) showing the number of steps taken (high score denotes good performance). **(B)** Placing pegs (F7CR105) showing the time taken to complete the task (low score denotes good performance). **(C)** Threading lace task (F7CR211) showing the time taken to complete the task (low score denotes good performance). **(D)** Throwing bean bag (F7CR331) showing the number of throws that successfully hit the target (high score denotes good performance).

Four components of the MABC (Henderson and Sugden, 1992) were identified as representative measures of motor coordination at age 7. The four tasks assess three aspects of coordination: balance (heel-to-toe walking), manual dexterity (placing pegs, threading lace), and ball skills (throwing bean bag into box). Heel-to-toe walking (F7CR015) measures the number of steps the child takes before stepping off a straight line. Throwing a beanbag (F7CR331) is the number of throws out of 10 where the beanbag is successfully thrown into a box using their preferred hand. Finally, the placing pegs task (F7CR105), inserting 12 pegs into a board, and the threading lace task (F7CR211), threading a lace through a series of holes in a board, are both measured as completion time. These raw scores for each individual measure were age-adjusted (Age in months at test), and the distributions of these individual measures are shown in Figure 1.

**FIGURE 2:**
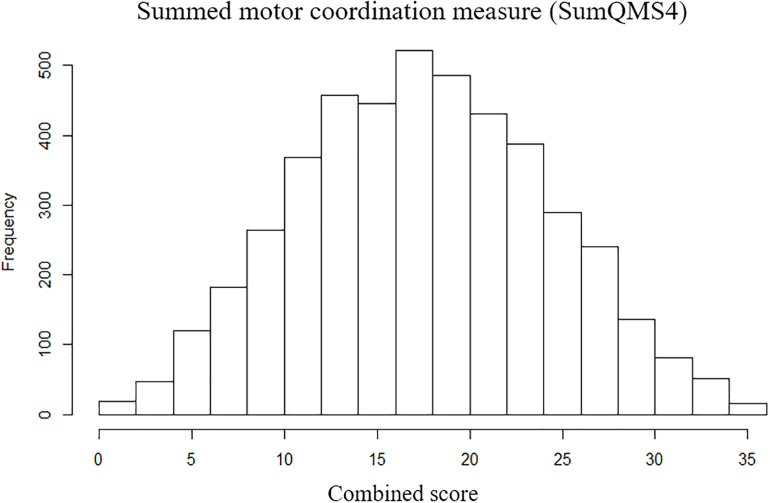
Distribution of summed motor coordination measures (SumQMS4) derived from the MABC test components to reflect an overall motor coordination score, and a maximum possible score of 40.

Scores were then expressed as percentile performance against the entire cohort. The placing pegs and threading lace scores were inverted so that for all scores a higher percentile denotes better performance. Each age-adjusted percentile score was transformed into an inverted point scale across the full range (such that individuals scoring between the 0 and 10th percentiles score 9, between the 10 and 20th percentiles score 8, and so on through to individuals between the 90 and 100th percentiles who score 0). Scores were summed across all four measures to generate a summed measure of overall motor coordination (SumQMS4) which was normally distributed (Figure 2) and ranged from 0 to 36 (mean 18.02, SD 6.71), where a higher score denotes worse performance. Note that although these measures spread across the full range of performance, the MABC was designed such that there will be a ceiling effect in typically developing children. As such, this test does not sensitively allow us to distinguish between children at the top of the motor skill range (Henderson et al., 2007).

In addition to the quantitative measure of motor ability, a binary measure of motor coordination was also derived. In this binary measure, each of the individual four motor measures were transformed to a point scale, this time focusing upon the lower tail of the distribution–the individuals with the poorest performance. Individuals scoring between the 0 and 2nd percentiles scored 5, between the 2 and 5th percentiles scored 4, between the 5 and 10th percentiles scored 3, between the 10 and 15th percentiles scored 2, between the 15 and 25th percentiles scored 1, and anyone above the 25th percentile scored 0, where again, a higher score denoted worse performance. Cases with motor coordination difficulties were defined as having a total summative score of ≥8 across all four tasks (a score that would require an average performance below the 10th percentile across all four tasks, *N* = 214). Controls were defined as individuals with score = 0 (≥25th percentile on all four tasks, *N* = 1737). The number of cases falling below this categorical cutoff was *N* = 214 or 5% of included children.

The available test battery does not allow us to unequivocally say that the children with motor coordination difficulties had DCD, as not all diagnostic criteria could be applied. We thus refer to this group as “probable” DCD (pDCD), allowing us to perform a case-control GWAS. Please note, this differs from the criteria used by Lingam et al. (2009) who also considered impact upon daily life as criteria for DCD and pDCD in accordance with the DSM-IV. All other individuals were excluded from analyses.

Graphs were plotted using the ggplot2 package^2^ (Wickham, 2016) within RStudio (v3.5.1).

### Genome Wide Association Study

The imputed genotype dataset contained 4,774,020 autosomal and X chromosome SNPs (chrs1-23) for the 4,542 individuals included in the study. A power calculation indicated that this sample provides 90.4% power to detect a variant that explains 1% of trait variance at a genome-wide threshold of significance and 99.0% power at a significance threshold of 1 × 10^–5^ [assuming a minor allele frequency (MAF) of 0.1, complete linkage disequilibrium (LD) between marker and causal variant]. This means we have sufficient power to detect common contributory variants that account for 1% of the population variation in motor control ability.

Standard quality control measures were performed on genome-wide SNP data prior to analysis (Anderson et al., 2010); SNPs with a minor allele frequency <5%, a per SNP call rate of <5%, a Hardy-Weinberg equilibrium *P* < 5 × 10^–7^ (*N* = 70) or a heterozygosity rate more than 3SD from the mean were excluded from analysis. Genotype rates were compared between motor difficulty cases and controls, and SNPs with a differential missing rate (*P* < 1 × 10^–5^, *N* = 4) were excluded from further analysis, leaving a total of 4,774,020 high quality SNPs. Individual genotype rate was checked, however, as this was an imputed dataset, all individuals had ≥95% coverage across SNPs.

One factor that can impact genetic association is differences in ancestry between individuals. To avoid this, genetic ethnicity was checked using a Principal Component Analysis (PCA) with the smartpca perl script^3^ from the Eigensoft package (Patterson et al., 2006; Price et al., 2006). These analyses compared a pruned set of low LD SNP data (95,225 variants) with the Human Genome Diversity Project (HGDP) dataset^4^ which includes seven populations of broad ethnicity (African, American, Central South Asia, East Asia, European, Middle East, and Oceania). The first and second Principal Components were plotted using the ggplot2 package (see footnote text 2) (Wickham, 2016) within RStudio (v3.5.1). All individuals appeared to be of European descent.

A set of 4,774,020 SNPs were analyzed for association using a general linear regression model within PLINK for the quantitative measure of coordination (SumQMS4), and a logistic model for the binary pDCD case/control phenotype. Genome-wide Manhattan and QQ plots were plotted in the qqman package (Turner, 2014) in RStudio (v3.5.1). Zoomed-in plots for suggestively associated loci were generated using Locus Zoom v0.12.0 (locuszoom.org). Genome-wide power calculation was performed using the online Genetic Power Calculator^5^.

## Results

### Quantitative Motor Coordination GWAS

**FIGURE 3:**
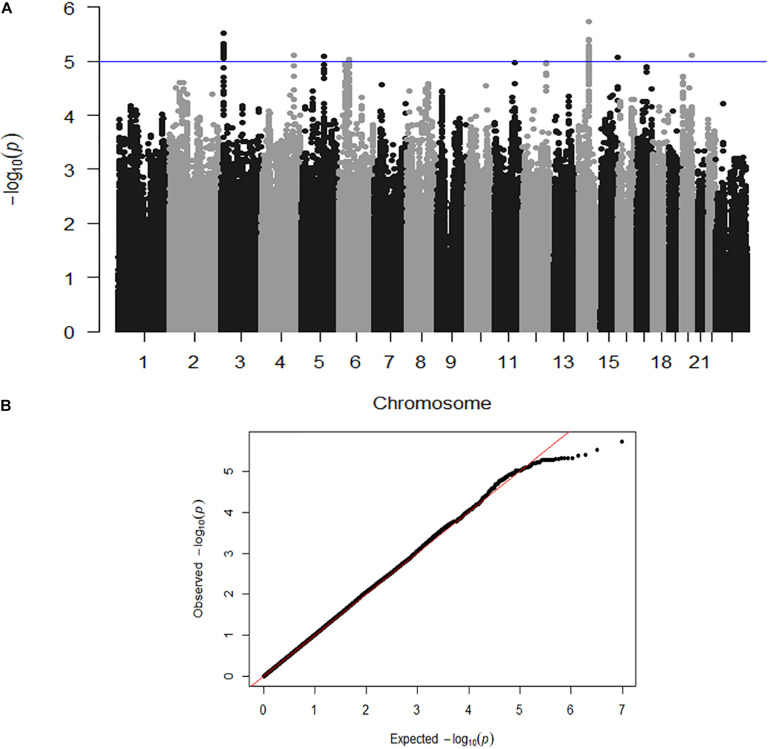
**(A)** Genome-wide association shown by a Manhattan plot indicating regions of nominal significance. Each point refers to a single genetic variation (SNP). The X-axis shows the position of SNPs along each of the 23 chromosomes. The Y axis shows the log-*P*-value indicating the strength of correlation between the genetic variant and the behavioral outcome (SumQMS4). **(B)** QQ plot showing the observed and expected association values are free from confounding population stratification.

Here we describe the first genome-wide association analysis of overall quantitative motor coordination (SumQMS4) in 4,542 children. This approach examines common genetic variants (SNPs) that are correlated with behavioral outcomes. No SNPs met genome-wide association (*P* ≤ 5 × 10^–8^), however, we identified 59 SNPs across seven genomic regions that met the threshold for suggestive significance at *P* ≤ 1 × 10^–5^ (Figure 3). These regions contain several potential genes of interest, that form potential pathways to investigate in future studies.

Figure 3A shows a Manhattan plot indicating suggestively associated regions on chromosomes 3, 4, 5, 6, 14, 15, and 20. Figure 3B shows the QQ plot of expected versus observed SNP association *P* values, indicating the absence of population stratification or other confounding variables.

Table 1 lists the seven chromosomal regions associated with SumQMS4, including flanking SNPs and genes. The full association results for all 59 top SNPs with *P* ≤ 5 × 10^–5^ can be found in the Supplementary materials (Supplementary Table 1).

**TABLE 1:** Motor coordination (SumQMS4) GWAS top-hits by regions.

**Position**	**Region**	**Flanking SNPs**	**No. SNPs**	**Min *P* value**	**Gene**	**Flanking genes**	**pDCD Case/control min *P* value**
chr3:13076226–13114852	3p25.2	rs11128630–rs62232913	16	3.07 × 10^–6^	*IQSEC1*	*–*	0.06095
chr4:157215618	4q32.1	rs17034349	1	7.90 × 10^–6^	*–*	*CTSO-PDGFC*	0.07066
chr5:110555735	5q22.1	rs75575712	1	8.05 × 10^–6^	*–*	*WDR36-CAMK4*	0.006457
chr6:53632969–53654299	6p12.1	rs9395876–rs4610551	8	9.17 × 10^–6^	*–*	*KLHL31-LRRC1*	0.4283
chr14:70875943–70935875	14q24.2	rs8012142–rs2293877	31	1.88 × 10^–6^	*SYNJ2BP, SYNJ2BP-COX16, ADAM21*, and *ADAM20P1*	*–*	0.01161
chr15:100783527	15q26.3	rs12324426	1	8.48 × 10^–6^	*ADAMTS17*	*–*	0.06136
chr20:51577026	20q13.2	rs2904292	1	7.78 × 10^–6^	–	*LINC01524-TSH7Z2*	0.03321

*The flanking SNPs, minimum *P* value are reported for each region. Genes contained within the associated region are listed, and in the case of intragenic regions we list flanking genes. The minimum *P* value from the corresponding region of the pDCD case/control GWAS is also listed (Bonferroni corrected *P* = 8.5 × 10^–4^).*

**FIGURE 4:**
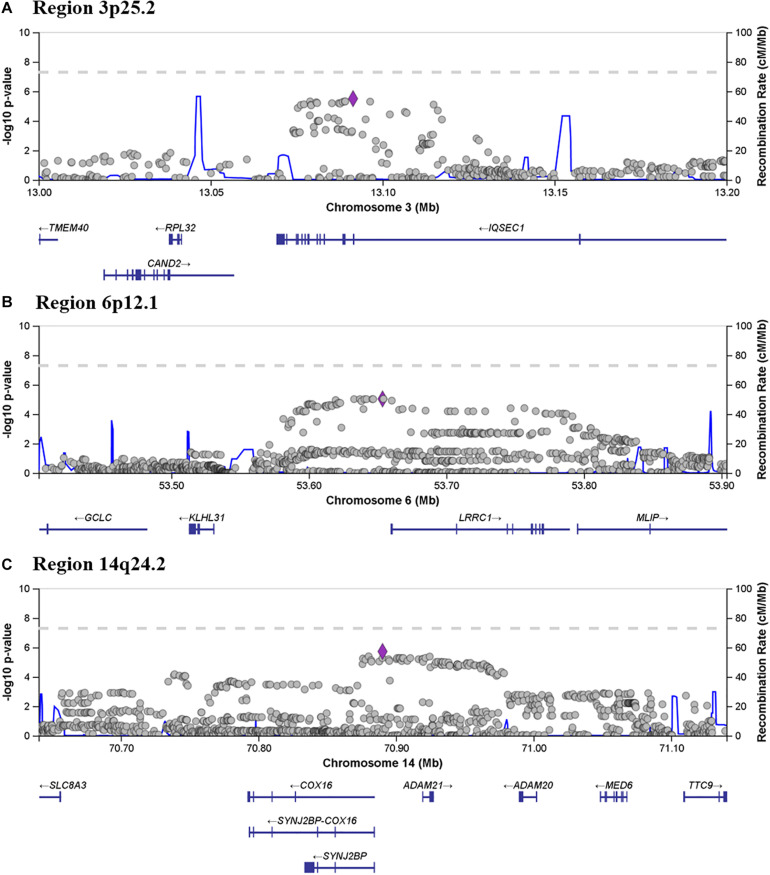
Locus zoom showing associated and flanking regions in **(A)** 3p25.2, **(B)** 6p12.1 and **(C)** 14q24.2, where more than one SNP met the criteria for suggestive association. Genes located within and flanking each of the three regions are indicated below each panel.

From the seven associated regions, three chromosome regions (3p25.2, 6p12.1, and 14q24.2) contained more than one suggestively statistically associated SNP, which is considered a marker of “true” association. Region 3p25.2 spans chr3:13076226–13114852 (rs11128630–rs62232913) and contains 16 SNPs with a *P* ≤ 8.34 × 10^–6^. Figure 4A shows a zoomed in view of the locus, showing that the association region lies within the gene *IQSEC1*. Region 6p12.1 spans region chr6:53632969–53654299 (rs9395876–rs4610551) and contains eight SNPs with a minimum *P* value of 9.43 × 10^–6^. The zoomed in locus view (Figure 4B) shows that this region of high association does not include any coding variants but is directly flanked by *LRRC1*. The most significantly associated SNP was rs8008210 (*P* = 1.88 × 10^–6^) which falls within the 14q24.2 region (chr14:70875943–70935875) (Figure 4C). This region contains 31 significant SNPs (rs8012142 to rs2293877) and overlaps fully with the entire *ADAM21* gene, the 3’ end of *ADAM20* and the 5’ of *SYNJ2BP* (Figure 4C).

### Association With pDCD Case Control

As the quantitative motor coordination score looks for association with motor coordination across the whole population regardless of ability, we then examined the top 59 most significant SNPs for association with the binary coordination difficulties status (pDCD cases = 214, controls = 1,737). The minimum *P* value associated with each region is reported in Table 1. None of the top 59 SNPs from the motor coordination GWAS were found to be associated with motor difficulty case-control status (Bonferroni correct *P* threshold is set to 8.5 × 10^–4^ to account for multiple testing). Individual association results for of the 59 top SNPs for the motor difficulties case/control association each can be found in Supplementary Table 1.

## Discussion

Here we report the first genetic association study of quantitative motor coordination in a population-based cohort of children. While no SNPs reached the threshold for genome-wide significance (*P* ≤ 5 × 10^–8^), three chromosome regions (3p25.2, 6p12.1, and 14q24.2) contained more than one suggestively associated SNP (*P* ≤ 1 × 10^–5^). The identification of a potential association of motor coordination with the genes *IQSEC1*, *LRRC1*, *SYNJ2BP*, *ADAM20*, and *ADAM21* do not offer a single clear potential molecular mechanism for motor coordination.

In a GWAS approach, we observe a statistical correlation between genetic variants and outcomes. This method does not provide information regarding the functional actions of those genetic variants. However, some information can be gained by considering the roles of the genes in which the associated variants fall. The 38Kb region on 3p25.2 (*P* ≤ 8.34 × 10^–6^) lies across the 5’ end of *IQSEC1*, including the 5’ untranslated region and coding exon 1. *IQSEC1* (OMIM ^∗^610166) also referred to as *BRAG2* or *GEP100*, the IQ motif and SEC7 domain containing-protein are involved in a wide range of cellular processes including cancer metastasis, angiogenesis, myoblast fusion and integrin trafficking (D’Souza and Casanova, 2016). IQSEC1 plays an important role in the structural organization and regulation of neurotransmitters present at the postsynaptic surface and is implicated in axon guidance through the Slit-robo pathway (Onel et al., 2004). Recessive, rare mutations in *IQSEC1* are associated with intellectual developmental disorder with short stature and behavioral abnormalities (OMIM #618687). Ansar et al. (2019) recently reported the first case of bi-allelic pathogenic variants in *IQSEC1*. Functional studies suggested that the pathogenic *IQSEC1* variants resulted in defects in axon guidance and dendritic projection processes. Affected family members presented with severe intellectual disability, short stature, speech aphasia and behavioral problems. Note that the variants described by Ansar differ from the common variations analyzed in the present study. The Ansar variants are rare pathogenic variations that have a clearly detrimental effect upon protein function, while the variants identified in the GWAS are common variants that may not directly impact protein coding, and are therefore expected to have only subtle effects on protein function. Interestingly, Ansar et al. reported that motor milestones were delayed, however, this is a common presentation in severe intellectual disability. Its known role in axon guidance and neurodevelopmental disorders make *IQSEC1* a plausible and interesting candidate for motor coordination, however, follow-up studies are necessary to validate this.

Interestingly, and perhaps relevant to a potential muscular process in DCD, *IQSEC1* is known to play a role in myoblast fusion, through the formation and repair of muscle (D’Souza and Casanova, 2016). Initially identified in fruit flies, and later confirmed in mammalian cell lines, *IQSEC1*/*BRAG2* knockouts showed impaired myoblast fusion (Chen et al., 2003). Although there is insufficient evidence for a direct effect, this highlights the potential for investigating underlying molecular mechanisms for DCD in model organisms.

The 21Kb region located on 6p12.1 (*P* ≤ 1 × 10^–6^) overlaps with the non-coding region immediately upstream of *LRRC1*, leucine rich repeat containing 1. *LRRC1* (OMIM ^∗^608195) is not reported to be associated with any genetic disorders. Research on the function of this gene is limited, however, it is reported to be involved in the Wnt/β-catenin signaling pathway, which is important in early development, and plays a role in cancer (Daulat et al., 2019).

The most significantly associated SNP fell within a 60Kb region within 14q24.2, overlapping with the 5’ region *SYNJ2BP*, the 3’ end of *ADAM20*, and the entirety of *ADAM21*. SYNJ2BP, synaptojanin 2 binding protein (OMIM ^∗^609411) is located on the outer membrane of the mitochondria (Hartmann et al., 2020) and forms an unusual gene read-through product with neighboring gene *COX16* (OMIM ^∗^618064) (Figure 4), a known mitochondrial complex IV assembly factor (Cerqua et al., 2018). Mitochondria play a vital role in brain function, and therefore this represents an interesting potential mechanism to further explore. On the other hand, *ADAM20* (OMIM ^∗^603712) and *ADAM21* (OMIM ^∗^603713), metallopeptidase domain 20 and 21, have not been previously reported to be associated with disease, and both are testes specific proteins (Hooft van Huijsduijnen, 1998).

Fliers et al. (2012) reported an enrichment of genes implicated in neurite outgrowth and muscle function in their GWAS of motor coordination measures in a cohort of children who met the criteria for ADHD. The genes and pathways identified in this study comprise a similar narrative - that genes with a known involvement in neuronal function are enriched. The total number of genes that were robustly associated in our study are too low to undertake a pathway analysis. The results of both studies indicate that neuronal mechanisms play a role in motor coordination, and that this is a promising area of future research.

Neither Fliers et al. (2012) nor the present study identified any SNPs that met the threshold for genome-wide significance. This is likely due to the relatively small sample sizes in both studies. As we could detect only a moderate effect size, far larger sample sizes are necessary to obtain statistically robust results. For example, a recent GWAS on schizophrenia successfully identified several novel and biologically validated regions of association (Schizophrenia Working Group of the Psychiatric Genomics Consortium., 2014). The success of this study is in part due to the sample size of more than 36,000 cases and 110,000 controls. The present study case/control analysis had only 214 cases and 1,737 controls, which meant that analyzing the derived quantitative measure of coordination (SumQMS4) provided more power than a case/control GWAS. Sample size is a major limitation of this study, and hence the findings should be considered as preliminary. Larger sample sizes, in the tens of thousands, are necessary for future GWAS, making meta-GWAS that combine multiple datasets a viable and potentially fruitful direction, although these approaches can present their own difficulties in terms of robust and consistent phenotype measurement.

The ALSPAC study contained a limited range of measures relating to motor coordination, however, the four components of the MABC are included in the current edition (MABC-2; Henderson et al., 2007) and are widely used for assessing and diagnosing motor coordination difficulties (Henderson and Sugden, 1992; Henderson et al., 2007; Blank et al., 2019). The complete MABC/MABC-2 is intended for identifying children with coordination difficulties and is not designed to accurately measure subtle differences in children within the typical range of ability. By deriving a composite measure of motor coordination (SumQMS4) using the four MABC elements, we were able to produce a general approximation of coordination ability at age 7 using the available data. Using a derived quantitative measure of coordination permitted us a far larger sample size (*N* = 4542) than a case-control analysis (motor difficulties case *N* = 214, controls *N* = 1737).

We are able to resolve the “tail” of children with coordination difficulties more reliably than those within the typical range. By defining the binary motor difficulties case group based on poor performance across multiple tests (<10th percentile), we defined an approximation for pDCD cases. It should be noted that this “case” definition is limited, and a full test battery is required to confidently diagnose DCD cases.

Lingam et al. (2009) used the same ALSPAC cohort to report the prevalence of DCD as 1.8%, and 2.23% met the criteria for probable DCD. The full available cohort (*N* = 7399) was included in their analysis as they were not limited to participants with available genotype data (*N* = 6500), prior to exclusions. Lingam et al. used three of the MABC elements (heel-to-toe walking, placing pegs and throwing a bean bag into a box) to represent each of the realms of coordination. Additional criteria (evidence that coordination difficulties impact their daily life) were applied to robustly define a DCD diagnosis in accordance with the DSM-IV definition. A DCD diagnosis was only applied if children had substantial handwriting difficulties at key stage 1 (age 7) and a parent reported difficulties with daily living. As in our study, Lingam et al. also excluded children with known non-developmental explanatory conditions (i.e., visual impairment, or medical condition), and children with mild intellectual disability (WISC FIQ < 70). In comparison, our inclusion criteria are more relaxed to allow us to capture motor difficulties more broadly, rather than a confident diagnosis of DCD or pDCD. While we recognize that using the additional life impact criteria would greatly improve confidence of diagnosis, it would have further reduced the already limited sample size, highlighting the need for balance between power and specificity.

Our results suggest a potential neuronal etiology to motor coordination difficulties, supported by the current literature (Licari et al., 2015; Blank et al., 2019; Brown-Lum et al., 2020). The identification of suggestive association of motor coordination with the genes *IQSEC1*, *LRRC1*, *SYNJ2BP*, *ADAM20*, and *ADAM21* do not offer a single clear potential molecular mechanism for motor coordination. Instead, it indicates *IQSEC1*, and the potential role of axon guidance and dendritic projection processes in motor coordination. This represents the most interesting candidate gene, although further validation would be necessary to understand the underlying mechanism.

Evidence that axonal development may be disrupted in children with DCD comes from a recent DTI study which compared the white matter structure of 31 children with a diagnosis of DCD to 30 neurotypical children (Brown-Lum et al., 2020). They found that children with DCD showed white matter differences in the corticospinal tract, posterior thalamic radiation, and the cerebellar pathways; all three of these regions have known roles in motor coordination. The low axial diffusivity observed in these areas are strongly suggestive of alterations in axonal structure and/or function within these regions (Brown-Lum et al., 2020). Brown-Lum et al. (2020) observed evidence of axonal changes in these regions, further supporting the potential role for axonal function in DCD.

Other neurodevelopmental disorders such as DLD provide a model for the genetic study of DCD and have uncovered underlying molecular mechanisms in related pathways. For example, Prasad et al. (2012) discovered a rare copy number variant in the gene *ROBO2* that was associated with ASD, while common variants in the same gene have been associated with expressive vocabulary development in infants (St Pourcain et al., 2014). The association of common variants in *ROBO2* with expressive vocabulary directly implicates the Slit-robo pathway and axon guidance as potential mechanisms for language development, and this mechanism is reflected in the association observed here with *IQSEC1*. A second example is the gene *FOXP2* in which some specific rare genetic variants result in a sub-type of language disorder called childhood apraxia of speech (CAS OMIM #602081) (Lai et al., 2001). In contrast to this specificity between genotype and phenotype, common variant within the *FOXP2* gene have been associated with both ADHD (Demontis et al., 2019; Soler Artigas et al., 2020) and intelligence (Lam et al., 2019) in GWAS studies. The role of rare and common variants specific to DLD and language disorders was comprehensively reviewed by Mountford and Newbury (2017).

It is highly likely that DCD will have a similar pattern to that seen in DLD, ASD, and other neurodevelopmental disorders, whereby there are both familial inherited variants that underlie specific phenotypes, and common variants in genes that contribute to motor coordination difficulties. The identification of these genes through a combination of family and large GWAS studies will contribute greatly to the underlying causes of DCD and help to support its recognition as an important neurodevelopmental disorder.

## Data Availability Statement

ALSPAC genotype data analyzed in this study are available upon application as outlined at http://www.bristol.ac.uk/alspac/researchers/access/. The ALSPAC website additionally contains details of all the data that is available through a fully searchable data dictionary and variable search tool (http://www.bristol.ac.uk/alspac/researchers/our-data/).

## Ethics Statement

Ethical approval for this study was obtained from Oxford Brookes University Research Ethics Committee (UREC #191311). Ethical approval for ALSPAC (B2341) was obtained from the ALSPAC Ethics and Law Committee and the Local Research Ethics Committees (http://www.bristol.ac.uk/alspac/researchers/research-ethics/). Informed consent for the use of data collected via questionnaires and clinics was obtained from participants following the recommendations of the ALSPAC Ethics and Law Committee at the time. Consent for biological samples has been collected in accordance with the Human Tissue Act (2004).

## Author Contributions

DN, AB, and HM designed and conceived the experiment. AH was the ALSPAC data buddy for this project and compiled and verified all ALSPAC datasets. DN and HM performed the genetic analyses. DN, HM, and AB wrote the manuscript. All authors contributed to the article and approved the submitted version.

## Conflict of Interest

The authors declare that the research was conducted in the absence of any commercial or financial relationships that could be construed as a potential conflict of interest.

## Footnotes

1
http://www.bristol.ac.uk/alspac/researchers/research-ethics/

2
https://ggplot2.tidyverse.org

3
https://github.com/chrchang/eigensoft/wiki/smartpca

4
https://www.internationalgenome.org/data-portal/data-collection/hgdp

5
https://zzz.bwh.harvard.edu/gpc

## Supplementary Material

Supplementary Table 1 Full list of association results for the 59 SNPs that met the threshold for suggestive association (*P* ≤ 5 × 10^–5^) on the SumQMS4. The statistical significance for each of the 59 SNPs is also reported for the binary DCD case/control association test.

## References

[B1] American Psychiatric Association (2013). *Diagnostic and Statistical Manual of Mental Disorders (DSM-5§) 1000 Wilson Boulevard, Arlington, VA 22209-3901*, 5th Edn. Washington, DC: American Psychiatric Association Publishing.

[B2] Anderson C. A. Pettersson F. H. Clarke G. M. Cardon L. R. Morris A. P. Zondervan K. T. (2010). Data quality control in genetic case-control association studies. *Nat. Protoc.* 5 1564–1573. 10.1038/nprot.2010.116 21085122 PMC3025522

[B3] Ansar M. Chung H. L. Al-Otaibi A. Elagabani M. N. Ravenscroft T. A. Paracha S. A. (2019). Bi-allelic variants in IQSEC1 cause intellectual disability, developmental delay, and short stature. *Am. J. Hum. Genet.* 105 907–920. 10.1016/j.ajhg.2019.09.013 31607425 PMC6848997

[B4] Barnett A. L. Prunty M. (2020). Handwriting difficulties in developmental coordination disorder (DCD). *Curr. Dev. Disord. Rep.* 8 6–14. 10.1007/s40474-020-00216-8

[B5] Becker N. Vasconcelos M. Oliveira V. Dos Santos F. C. Bizarro L. De Almeida R. M. M. (2017). Genetic and environmental risk factors for developmental dyslexia in children: systematic review of the last decade. *Dev. Neuropsychol.* 42 423–445. 10.1080/87565641.2017.1374960 29068706

[B6] Bishop D. V. M. (2010). Which neurodevelopmental disorders get researched and why? *PLoS One* 5:e15112. 10.1371/journal.pone.0015112 21152085 PMC2994844

[B7] Blank R. Barnett A. L. Cairney J. Green D. Kirby A. Polatajko H. (2019). International clinical practice recommendations on the definition, diagnosis, assessment, intervention, and psychosocial aspects of developmental coordination disorder. *Dev. Med. Child Neurol.* 61 242–285. 10.1111/dmcn.14132 30671947 PMC6850610

[B8] Boyd A. Golding J. Macleod J. Lawlor D. A. Fraser A. Henderson J. (2013). Cohort Profile: the ‘children of the 90s’–the index offspring of the Avon Longitudinal Study of Parents and Children. *Int. J. Epidemiol.* 42 111–127. 10.1093/ije/dys064 22507743 PMC3600618

[B9] Brown-Lum M. Izadi-Najafabadi S. Oberlander T. F. Rauscher A. Zwicker J. G. (2020). Differences in white matter microstructure among children with developmental coordination disorder. *JAMA Network Open* 3:e201184. 10.1001/jamanetworkopen.2020.1184 32186744 PMC7081126

[B10] Cairney J. Hay J. A. Faught B. E. Hawes R. (2005). Developmental coordination disorder and overweight and obesity in children aged 9-14 y. *Int. J. Obes.* 29 369–372. 10.1038/sj.ijo.0802893 15768042

[B11] Cantell M. H. Smyth M. M. Ahonen T. P. (1994). Clumsiness in adolescence: educational, motor, and social outcomes of motor delay detected at 5 years. *Adapt. Phys. Activ. Q.* 11 115–129. 10.1123/apaq.11.2.115

[B12] Cerqua C. Morbidoni V. Desbats M. A. Doimo M. Frasson C. Sacconi S. (2018). COX16 is required for assembly of cytochrome c oxidase in human cells and is involved in copper delivery to COX2. *Biochim. Biophys. Acta Bioenerg.* 1859 244–252. 10.1016/j.bbabio.2018.01.004 29355485

[B13] Chen E. H. Pryce B. A. Tzeng J. A. Gonzalez G. A. Olson E. N. (2003). Control of myoblast fusion by a guanine nucleotide exchange factor, loner, and its effector ARF6. *Cell* 114 751–762. 10.1016/s0092-8674(03)00720-7 14505574

[B14] Cleaton M. A. M. Lorgelly P. K. Kirby A. (2019). Developmental coordination disorder: the impact on the family. *Qual. Life Res.* 28 925–934. 10.1007/s11136-018-2075-1 30536221

[B15] Coe B. P. Witherspoon K. Rosenfeld J. A. van Bon B. W. Vulto-van Silfhout A. T. Bosco P. (2014). Refining analyses of copy number variation identifies specific genes associated with developmental delay. *Nat. Genet.* 46 1063–1071. 10.1038/ng.3092 25217958 PMC4177294

[B16] Cunningham A. C. Hall J. Owen M. J. van den Bree M. B. M. (2019). Coordination difficulties, IQ and psychopathology in children with high-risk copy number variants. *Psychol. Med.* 51 290–299. 10.1017/S0033291719003210 31739810 PMC7234895

[B17] Daulat A. M. Wagner M. S. Walton A. Baudelet E. Audebert S. Camoin L. (2019). The tumor suppressor SCRIB is a negative modulator of the Wnt/beta-catenin signaling pathway. *Proteomics* 19:e1800487. 10.1002/pmic.201800487 31513346

[B18] Delaneau O. Zagury J. Marchini J. (2013). Improved whole-chromosome phasing for disease and population genetic studies. *Nat. Methods* 10 5–6. 10.1038/nmeth.2307 23269371

[B19] Demontis D. Walters R. K. Martin J. Mattheisen M. Als T. D. Agerbo E. (2019). Discovery of the first genome-wide significant risk loci for attention deficit/hyperactivity disorder. *Nat. Genet.* 51 63–75. 10.1038/s41588-018-0269-7 30478444 PMC6481311

[B20] D’Souza R. S. Casanova J. E. (2016). The BRAG/IQSec family of Arf GEFs. *Small GTPases* 7 257–264. 10.1080/21541248.2016.1219442 27739918 PMC5129896

[B21] Fedorenko E. Morgan A. Murray E. Cardinaux A. Mei C. Tager-Flusberg H. (2016). A highly penetrant form of childhood apraxia of speech due to deletion of 16p11.2. *Eur. J. Hum. Genet.* 24 302–306. 10.1038/ejhg.2015.149 26173965 PMC4717199

[B22] Fliers E. A. Vasquez A. A. Poelmans G. Rommelse N. Altink M. Buschgens C. (2012). Genome-wide association study of motor coordination problems in ADHD identifies genes for brain and muscle function. *World J. Biol. Psychiatry* 13 211–222. 10.3109/15622975.2011.560279 21473668

[B23] Fraser A. Macdonald-Wallis C. Tilling K. Boyd A. Golding J. Davey Smith G. (2013). Cohort profile: the avon longitudinal study of parents and children: ALSPAC mothers cohort. *Int. J. Epidemiol.* 42 97–110. 10.1093/ije/dys066 22507742 PMC3600619

[B24] Gaines R. Collins D. Boycott K. Missiuna C. DeLaat D. Soucie H. (2008). Clinical expression of developmental coordination disorder in a large Canadian family. *Paediatr. Child Health* 13 763–768. 10.1093/pch/13.9.763 19436536 PMC2603148

[B25] Harrowell I. Hollén L. Lingam R. Emond A. (2018). The impact of developmental coordination disorder on educational achievement in secondary school. *Res. Dev. Disabil.* 72 13–22. 10.1016/j.ridd.2017.10.014 29080482 PMC5770330

[B26] Hartmann C. Schwietzer Y. A. Kummer D. Kirschnick N. Hoppe E. Thuring E. M. (2020). The mitochondrial outer membrane protein SYNJ2BP interacts with the cell adhesion molecule TMIGD1 and can recruit it to mitochondria. *BMC Mol. Cell Biol.* 21:30. 10.1186/s12860-020-00274-1 32303178 PMC7164261

[B27] Henderson S. E. Sugden D. A. (1992). *Movement Assessment Batteryfor Children; Manual.* Sidcup: The Psychological Corporation.

[B28] Henderson S. E. Sugden D. A. Barnett A. L. (2007). *Movement Assessment Battery for Children–Second Edition.* London: Harcourt Assessmen.

[B29] Hooft van Huijsduijnen R. (1998). ADAM 20 and 21; two novel human testis-specific membrane metalloproteases with similarity to fertilin-alpha. *Gene* 206 273–282. 10.1016/s0378-1119(97)00597-0 9469942

[B30] Kalnak N. Stamouli S. Peyrard-Janvid M. Rabkina I. Becker M. Klingberg T. (2018). Enrichment of rare copy number variation in children with developmental language disorder. *Clin. Genet.* 94 313–320. 10.1111/cge.13389 29851021

[B31] Kirby A. Edwards L. Sugden D. A. (2011). Emerging adulthood in developmental co-ordination disorder: parent and young adult perspectives. *Res. Dev. Disabil.* 32 1351–1360. 10.1016/j.ridd.2011.01.041 21334175

[B32] Kirby A. Sugden D. A. Beveridge S. Edwards L. (2008). Developmental co-ordination disorder (DCD) in adolescents and adults in further and higher education. *J. Res. Special Educ. Needs* 8 120–131. 10.1111/j.1471-3802.2008.00111.x

[B33] Lai C. S. Fisher S. E. Hurst J. A. Vargha-Khadem F. Monaco A. P. (2001). A forkhead-domain gene is mutated in a severe speech and language disorder. *Nature* 413 519–523. 10.1038/35097076 11586359

[B34] Lam M. Hill W. D. Trampush J. W. Yu J. Knowles E. Davies G. (2019). Pleiotropic meta-analysis of cognition, education, and schizophrenia differentiates roles of early neurodevelopmental and adult synaptic pathways. *Am. J. Hum. Genet.* 105 334–350. 10.1016/j.ajhg.2019.06.012 31374203 PMC6699140

[B35] Larsen R. F. Mortensen L. H. Martinussen T. Andersen A. M. N. (2013). Determinants of developmental coordination disorder in 7-year-old children: a study of children in the Danish National Birth Cohort. *Dev. Med. Child Neurol.* 55 1016–1022. 10.1111/dmcn.12223 23909795

[B36] Licari M. K. Billington J. Reid S. L. Wann J. P. Elliott C. M. Winsor A. M. (2015). Cortical functioning in children with developmental coordination disorder: a motor overflow study. *Exp. Brain Res.* 233 1703–1710. 10.1007/s00221-015-4243-7 25757959

[B37] Lichtenstein P. Carlström E. Råstam M. Gillberg C. Anckarsäter H. (2010). The genetics of autism spectrum disorders and related neuropsychiatric disorders in childhood. *Am. J. Psychiatry* 167 1357–1363. 10.1176/appi.ajp.2010.10020223 20686188

[B38] Lingam R. Golding J. Jongmans M. J. Hunt L. P. Ellis M. Emond A. (2010). The association between developmental coordination disorder and other developmental traits. *Pediatrics* 126 e1109–e1118. 10.1542/peds.2009-2789 20956425

[B39] Lingam R. Hunt L. Golding J. Jongmans M. Emond A. (2009). Prevalence of developmental coordination disorder using the DSM-IV at 7 years of age: a UK population–based study. *Pediatrics* 123 e693–e700. 10.1542/peds.2008-1770 19336359

[B40] Lionel A. C. Crosbie J. Barbosa N. Goodale T. Thiruvahindrapuram B. Rickaby J. (2011). Rare copy number variation discovery and cross-disorder comparisons identify risk genes for ADHD. *Sci. Transl. Med.* 3:95ra75. 10.1126/scitranslmed.3002464 21832240

[B41] Losse A. Henderson S. E. Elliman D. Hall D. Knight E. Jongmans M. (1991). Clumsiness in children-do they grow out of it? A 10-year follow-up study. *Dev. Med. Child Neurol.* 33 55–68. 10.1111/j.1469-8749.1991.tb14785.x 1704864

[B42] Martin N. C. Piek J. P. Hay D. (2006). DCD and ADHD: a genetic study of their shared aetiology. *Hum. Mov. Sci.* 25 110–124. 10.1016/j.humov.2005.10.006 16442650

[B43] Moruzzi S. Pesenti-Gritti P. Brescianini S. Salemi M. Battaglia M. Ogliari A. (2010). Clumsiness and psychopathology: causation or shared etiology? A twin study with the CBCL 6–18 questionnaire in a general school-age population sample. *Hum. Mov. Sci.* 29 326–338. 10.1016/j.humov.2010.01.005 20338654

[B44] Mosca S. J. Langevin L. M. Dewey D. Innes A. M. Lionel A. C. Marshall C. C. (2016). Copy-number variations are enriched for neurodevelopmental genes in children with developmental coordination disorder. *J. Med. Genet.* 53 812–819. 10.1136/jmedgenet-2016-103818 27489308

[B45] Mountford H. S. Newbury D. F. (2017). The genomic landscape of language: insights into evolution. *J. Lang. Evol.* 3 49–58. 10.1093/jole/lzx019

[B46] Mountford H. S. Bishop D. V. M. Thompson P. A. Simpson N. H. Newbury D. F. (2020). Copy number variation burden does not predict severity of neurodevelopmental phenotype in children with a sex chromosome trisomy. *Am. J. Med. Genet. Part C* 184 256–266. 10.1002/ajmg.c.31791 32452638

[B47] Newbury D. F. Mari F. Sadighi Akha E. Macdermot K. D. Canitano R. Monaco A. P. (2013). Dual copy number variants involving 16p11 and 6q22 in a case of childhood apraxia of speech and pervasive developmental disorder. *Eur. J. Hum. Genet.* 21 361–365. 10.1038/ejhg.2012.166 22909776 PMC3598310

[B48] Onel S. Bolke L. Klambt C. (2004). The *Drosophila* ARF6-GEF Schizo controls commissure formation by regulating Slit. *Development* 131 2587–2594. 10.1242/dev.01147 15148300

[B49] Patterson N. Price A. L. Reich D. (2006). Population structure and eigenanalysis. *PLoS Genet.* 2:e190. 10.1371/journal.pgen.0020190 17194218 PMC1713260

[B50] Prasad A. Merico D. Thiruvahindrapuram B. Wei J. Lionel A. C. Sato D. (2012). A discovery resource of rare copy number variations in individuals with autism spectrum disorder. *G3* 2 1665–1685. 10.1534/g3.112.004689 23275889 PMC3516488

[B51] Price A. L. Patterson N. J. Plenge R. M. Weinblatt M. E. Shadick N. A. Reich D. (2006). Principal components analysis corrects for stratification in genome-wide association studies. *Nat. Genet.* 38 904–909. 10.1038/ng1847 16862161

[B52] Sanders S. J. He X. Willsey A. J. Ercan-Sencicek A. G. Samocha K. E. Cicek A. E. (2015). Insights into autism spectrum disorder genomic architecture and biology from 71 risk loci. *Neuron* 87 1215–1233. 10.1016/j.neuron.2015.09.016 26402605 PMC4624267

[B53] Schizophrenia Working Group of the Psychiatric Genomics Consortium. (2014). Biological insights from 108 schizophrenia-associated genetic loci. *Nature* 511 421–427. 10.1038/nature13595 25056061 PMC4112379

[B54] Simpson N. H. Ceroni F. Reader R. H. Covill L. E. Knight J. C. Consortium S. L. I. (2015). Genome-wide analysis identifies a role for common copy number variants in specific language impairment. *Eur. J. Hum. Genet.* 23 1370–1377. 10.1038/ejhg.2014.296 25585696 PMC4592089

[B55] Soler Artigas M. Sanchez-Mora C. Rovira P. Richarte V. Garcia-Martinez I. Pagerols M. (2020). Attention-deficit/hyperactivity disorder and lifetime cannabis use: genetic overlap and causality. *Mol. psychiatry* 25 2493–2503. 10.1038/s41380-018-0339-3 30610198 PMC8025199

[B56] St Pourcain B. Cents R. A. Whitehouse A. J. Haworth C. M. Davis O. S. O’Reilly P. F. (2014). Common variation near ROBO2 is associated with expressive vocabulary in infancy. *Nat. Commun.* 5:4831. 10.1038/ncomms5831 25226531 PMC4175587

[B57] Stephenson E. A. Chesson R. A. (2008). ‘Always the guiding hand’: parents’ accounts of the long-term implications of developmental co-ordination disorder for their children and families. *Child* 34 335–343. 10.1111/j.1365-2214.2007.00805.x 18410640

[B58] Summers J. Larkin D. Dewey D. (2008). Activities of daily living in children with developmental coordination disorder: dressing, personal hygiene, and eating skills. *Hum. Mov. Sci.* 27 215–229. 10.1016/j.humov.2008.02.002 18348898

[B59] Tsiotra G. D. Flouris A. D. Koutedakis Y. Faught B. E. Nevill A. M. Lane A. M. (2006). A comparison of developmental coordination disorder prevalence rates in Canadian and Greek children. *J. Adolesc. Health* 39 125–127. 10.1016/j.jadohealth.2005.07.011 16781974

[B60] Turner S. D. (2014). qqman: an R package for visualizing GWAS results using Q-Q and manhattan plots. *BioRxiv* [preprint] 10.1101/005165005165.

[B61] Van Waelvelde H. De Weerdt W. De Cock P. Smits-Engelsman B. C. (2004). Aspects of the validity of the movement assessment battery for children. *Hum. Mov. Sci.* 23 49–60. 10.1016/j.humov.2004.04.004 15201041

[B62] Wickham H. (2016). *ggplot2: Elegant Graphics for Data Analysis.* New York, NY: Springer-Verlag New York.

[B63] Wright H. C. Sugden D. A. (1996). A two-step procedure for the identification of children with developmental co-ordination disorder in Singapore. *Dev. Med. Child Neurol.* 38 1099–1105. 10.1111/j.1469-8749.1996.tb15073.x 8973295

