# Corrigendum: Genome-wide association study of motor coordination

**DOI:** 10.3389/fnhum.2024.1360116

**Published:** 2024-01-23

**Authors:** Hayley S. Mountford, Amanda Hill, Anna L. Barnett, Dianne F. Newbury

**Affiliations:** ^1^Department of Biological and Medical Sciences, Faculty of Health and Life Sciences, Oxford Brookes University, Oxford, United Kingdom; ^2^Population Health Sciences, Bristol Medical School, University of Bristol, Bristol, United Kingdom; ^3^Centre for Psychological Research, Faculty of Health and Life Sciences, Oxford Brookes University, Oxford, United Kingdom

**Keywords:** coordination, development, dyspraxia, neurodevelopment, GWAS, ALSPAC, developmental coordination disorder, motor coordination

In the published article, there was an error. The genotypes used in the study were imputed to 1000 Genomes v1.3, not to Haplotype Reference Consortium (HRC) panel (pre-release 2015) as stated.

A correction has been made to Methods, *Avon longitudinal study of parents and children population cohort*, paragraph number three. This sentence previously stated:

“These data were jointly phased using SHAPEIT2 (Delaneau et al., 2013), which uses relationship information to improve phasing accuracy, and imputed to the Haplotype Reference Consortium (HRC) panel (pre-release 2015)”.

The corrected sentence appears below:

“These data were jointly phased using SHAPEIT2 (Delaneau et al., 2013), which uses relationship information to improve phasing accuracy, and imputed to the 1,000 Genomes v1.3.”

The authors apologize for this error and state that this does not change the scientific conclusions of the article in any way. The original article has been updated.
